# Optical genome mapping identifies clinically relevant genomic rearrangements in prostate cancer biopsy sample

**DOI:** 10.1186/s12935-022-02728-2

**Published:** 2022-10-08

**Authors:** Yeeun Shim, Jongsoo Lee, Jieun Seo, Cheol Keun Park, Saeam Shin, Hyunho Han, Seung-Tae Lee, Jong Rak Choi, Byung Ha Chung, Young Deuk Choi

**Affiliations:** 1 Department of Laboratory Medicine, Graduate School of Medical Science, Brain Korea 21 project, Yonsei University College of Medicine, Seoul, Republic of Korea; 2 Department of Urology, Urological Science Institute, Yonsei University College of Medicine, Seoul, Republic of Korea; 3 Department of Genetics, School of Medicine, Washington University in St. Louis, St. Louis, MO 63110 USA; 4 Department of Pathology, Yonsei University College of Medicine, Seoul, Republic of Korea; 5 Pathology Center, Seegene Medical Foundation, Seoul, Republic of Korea; 6 Department of Laboratory Medicine, Yonsei University College of Medicine, Seoul, Republic of Korea; 7 Dxome Seongnam-daero 331 beon-gil, Bundang-gu, Seongnam-si Gyeonggi-do, Republic of Korea

**Keywords:** Prostate cancer, Optical genome mapping, Structural variation, Genomic rearrangement, *BRCA2*

## Abstract

**Background:**

Prostate cancer (PCa) is characterized by complex genomic rearrangements such as the ETS oncogene family fusions, yet the clinical relevance is not well established. While paneled genetic tests of DNA repair genes are recommended in advanced PCa, conventional genomic or cytogenetic tools are not ideal for genome-wide screening of structural variations (SVs) such as balanced translocation due to cost and/or resolution issues.

**Methods:**

In this study, we tested the feasibility of whole-genome optical genomic mapping (OGM), a newly developed platform for genome-wide SV analysis to detect complex genomic rearrangements in consecutive unselected PCa samples from MRI/US-fusion targeted biopsy.

**Results:**

We tested ten samples, and nine (90%) passed quality check. Average mapping rate and coverage depth were 58.1 ± 23.7% and 157.3 ± 97.7×, respectively (mean ± SD). OGM detected copy number alterations such as chr6q13 loss and chr8q12-24 gain. Two adjacent tumor samples were distinguished by inter/intra-chromosomal translocations, revealing that they’re from the same ancestor. Furthermore, OGM detected large deletion of chr13q13.1 accompanied by inter-chromosomal translocation t(13;20)(q13.1;p13) occurring within *BRCA2* gene, suggesting complete loss of function.

**Conclusion:**

In conclusion, clinically relevant genomic SVs were successfully detected in PCa samples by OGM. We suggest that OGM can complement panel sequencing of DNA repair genes *BRCA1/2* or *ATM* in high-risk PCa.

**Supplementary Information:**

The online version contains supplementary material available at 10.1186/s12935-022-02728-2.

## Background

Prostate cancer (PCa) genome is characterized by a relatively low mutation burden but high frequency of large complex structural variations (SVs) [1, 2]. For example, ETS family gene fusion is a known driver of the disease and occurs by intra- or inter- chromosomal translocations or deletions [3]. Thereby, genome-wide SV analysis of PCa can provide valuable information regarding its pathogenesis [4]. Current modalities of genome-wide SV analysis include karyotyping, fluorescence in situ hybridization (FISH), chromosomal microarrays (CMA), and recent addition of whole-genome sequencing (WGS). Yet, each tool has its own limitation (resolution, manual labor, requirement of live cells, inability to detect balanced translocation or cost) and is not wildly applied to study complex SVs in prostate cancer samples. In fact, genome-wide SV analysis for PCa is not popular in clinic yet.

Germline and somatic mutations of DNA repair genes such as *BRCA1, BRCA2*, and *ATM* in PCa have been associated with aggressive disease course and resistance to androgen receptor-targeted therapies [5–7]. Recently, PARP inhibitors have improved survival in PCa patients with DNA repair gene mutations, particularly *BRCA2* [8, 9]. In this regard, precise assessment of *BRCA2* and other DNA repair genes is now recommended for all metastatic PCa patients [10].

Optical genome mapping (OGM) is a recently developed genomic SV detection tool with superior sensitivity to conventional cytogenetic methods [11], particularly for the detection of balanced rearrangement. It can aid a sequencing-based approach to detect clinically relevant genomic aberrations and characterize complex SVs such as chromoplexy or chromothripsis [12–14]. Also, it has provided results comparable to conventional cytogenetic techniques in diagnosis and prognostic genomic marker detection of leukemia and multiple myeloma [15, 16].

In this study, we performed OGM on PCa tissues acquired by transrectal ultrasonography (US)-guided needle biopsy on target lesion from MRI at the time of diagnosis. OGM found clinically relevant SVs affecting tumor suppressor genes such as *BRCA2* and genomic heterogeneity between two adjacent tumors.

## Materials and methods

### Patient samples

The study was approved by the Bioethics Committee of the Severance Hospital (No. 4-2021-0276). All participating individuals gave informed consent.

### Prostate cancer histology

We used the 2014 Gleason grade group (GG) proposed by the ISUP. Gleason grade is a point 1 to 5 given to the histological findings of prostate adenocarcinoma - grade 1 as well-differentiated to grade 5 as poorly differentiated. Gleason grade can vary in a single patient across the tumor regions. Gleason score is a sum of the most commonly observed Gleason grade and the second-most commonly observed grade. For instance, Gleason score 9 (5 + 4) means that the most commonly observed Gleason grade is 5, and the second-most commonly observed grade is 4 in a tumor. The 2014 ISUP Gleason grade group (GG) is a five-tier prognostic categorization of the Gleason score into five groups. GG1 - Gleason score 6(3 + 3); GG2: Gleason score 7(3 + 4); GG3: Gleason score 7(4 + 3); GG4: Gleason score 8(4 + 4); GG5: Gleason score 9(4 + 5), 9(5 + 4) or 10(5 + 5).

### Ultra-High Molecular Weight (UHMW) gDNA isolation from frozen tissue

Ten prostate cancer tissues were collected from prostate needle biopsy. The tissue was snap-frozen and stored at − 80℃. ~10 mg frozen tissue was used for ultra-high molecular weight (UHMW) gDNA following the tissue and tumor DNA isolation protocol (#30,339) from Bionano Genomics (https://bionanogenomics.com/support-page/sp-tissue-and-tumor-dna-isolation-kit/). The tissue was mechanically homogenized in a buffer containing ethanol and filtered through 40 μm cell strainer. Tissue homogenate was pelleted by centrifugation. Resuspended pellets were lysed and digested, then Phenylmethylsulfonyl Fluoride Solution (PMSF, Millipore Sigma) was added to inactivate Proteinase K. Released gDNA binds to a single paramagnetic Nanobind Disk (Bionano Genomics, San Diego, CA, USA) following addition of salting buffer (SB, Bionano Genomics) and 100% isopropanol (Fisher Scientific, Hampton, NH, USA). After four wash steps, the disk was transferred to a clean tube, and the gDNA was eluted at room temperature.

### Direct label and staining (DLS)

Using Bionano Prep Direct Label and Stain assay (#30,206, https://bionanogenomics.com/support-page/dna-labeling-kit-dls/), 750 ng UHMW gDNA was enzymatically labeled at a specific sequence motif (green). Proteinase K (Qiagen, Hilden, Germany) was used to inactivate DLE-1 enzyme. After a series of membrane adsorption, the labeled DNA was stained for backbone visualization (blue). All procedures were done according to the manufacturer’s instructions.

Labeled UHMW gDNA was quantified by Qubit dsDNA High Sensitivity Assay (Thermo Fisher Scientic, Waltham, MA, USA) and 4–12 ng/µL labeled samples were directly loaded onto Bionano Saphyr® Chip G2.3.

### Bionano Solve Pipeline

Genome map assembly, alignments, and structural variation calling were generated using Bionano Solve v3.5. Genome Reference Consortium Human Build 37 (GRCh37) was used for the reference genome. Analyzed data was visualized with Bionano Access v1.5.2.

## Results

**Fig. 1:**
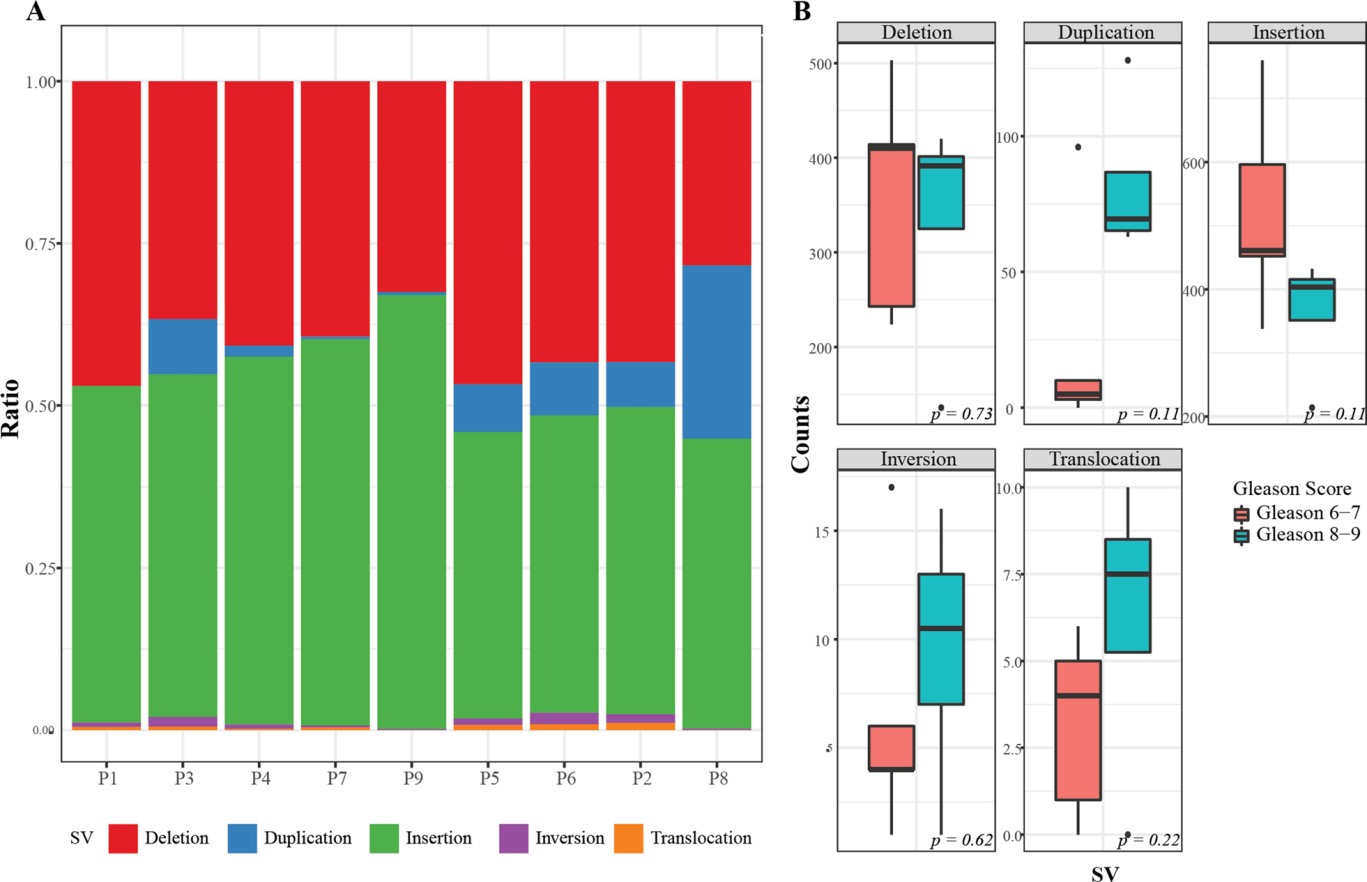
Component of Structural Variations in Prostate Cancer Tissue Sample. **A** Stacked bar plot of structural variation types across samples. **B** Comparison of structural variation types between Gleason grade group 1–2 tumors and Gleason grade group 4–5 tumors. *p-*value by Wilcoxon signed-rank test

We performed OGM of ten prostate needle biopsy samples, and nine samples passed the initial quality check. One sample could not be included in this study due to insufficient DNA concentration. Clinical characteristics of the tumors are summarized in Table 1. N50 range of the analyzed DNA molecules were 195.4 to 235.9 Kbp. The average mapping rate and coverage depth was 58.1 ± 23.7% and 157.3 ± 97.7×, respectively (mean ± SD) (Additional file 2: Table S1). When aligned to reference human genome (GRCh37), 479 to 1,227 SVs (mean ± SD: 868 ± 236) were found. The most frequent SV type was insertion, followed by deletion (Table 2; Fig. 1A). When divided by histologic grades (low Gleason grade group (GG): 1–3, n = 5; high GG: 4–5, n = 4), high GG tumors showed more frequent duplications, inversions, and translocations than low GG tumors, yet not statistically significant (Fig. 1B, p-value by Wilcoxon rank test).

**Table 1:** Summary of 9 prostate cancer samples

Sample name	Age	MRI findings	PSA (ng/mL)	Target lesion Gleason Score	ISUP GG
P1	68	Left anterior TZ, PI-RADS5, cT3aN0	5.79	6 (3 + 3)	1
P2	86	Right midgland PZ, PI-RADS5, cT3aN0	6.36	9 (5 + 4)	5
P3	83	Right midgland PZ, PI-RADS5, cT2bN0	18	7 (3 + 4)	2
P4	63	Left TZ, PI-RADS5, cT2bN0	24	7 (3 + 4)	2
P5*	78	Right TZ, PI-RADS5, cT3aN0	16.9	8 (4 + 4)	4
P6*	78	Right TZ, PI-RADS5, cT3aN0	16.9	8 (4 + 4)	4
P7	80	A large tumor involving both TZ, PI-RADS5, cT2cN0	43.9	7 (3 + 4)	2
P8	78	Multifocal, diffuse lesion involving both PZ and right TZ, PI-RADS5, cT3aN1	30.7	9 (5 + 4)	5
P9	62	Right PZ, PI-RADS5, cT3N0	76.7	7 (4 + 3)	3

*MRI* magnetic resonance imaging,* PSA* prostate specific antigen,* ISUP GG* International Society of Urological Pathology Gleason grade group,* TZ* transition zone,* PI-RADS* Prostate Imaging–Reporting and Data System,* PZ* peripheral zone

*From the same patient

**Table 2:** Structural variation summary

	P1	P2	P3	P4	P5	P6	P7*	P8	P9*
Deletions (> 1 kb, < 1 Mb)	410	395	414	243	420	388	503	136	224
Insertions (> 1 kb)	452	432	596	338	397	410	760	214	461
Duplications	63	63	96	10	66	73	5	128	3
Inversion	6	12	17	4	9	16	4	1	1
Inter-chromosomal translocations	1	1	3	0	2	3	4	0	0
Intra-chromosomal translocations	3	9	3	1	5	5	1	0	0

*De novo assembly pipeline

**Fig. 2:**
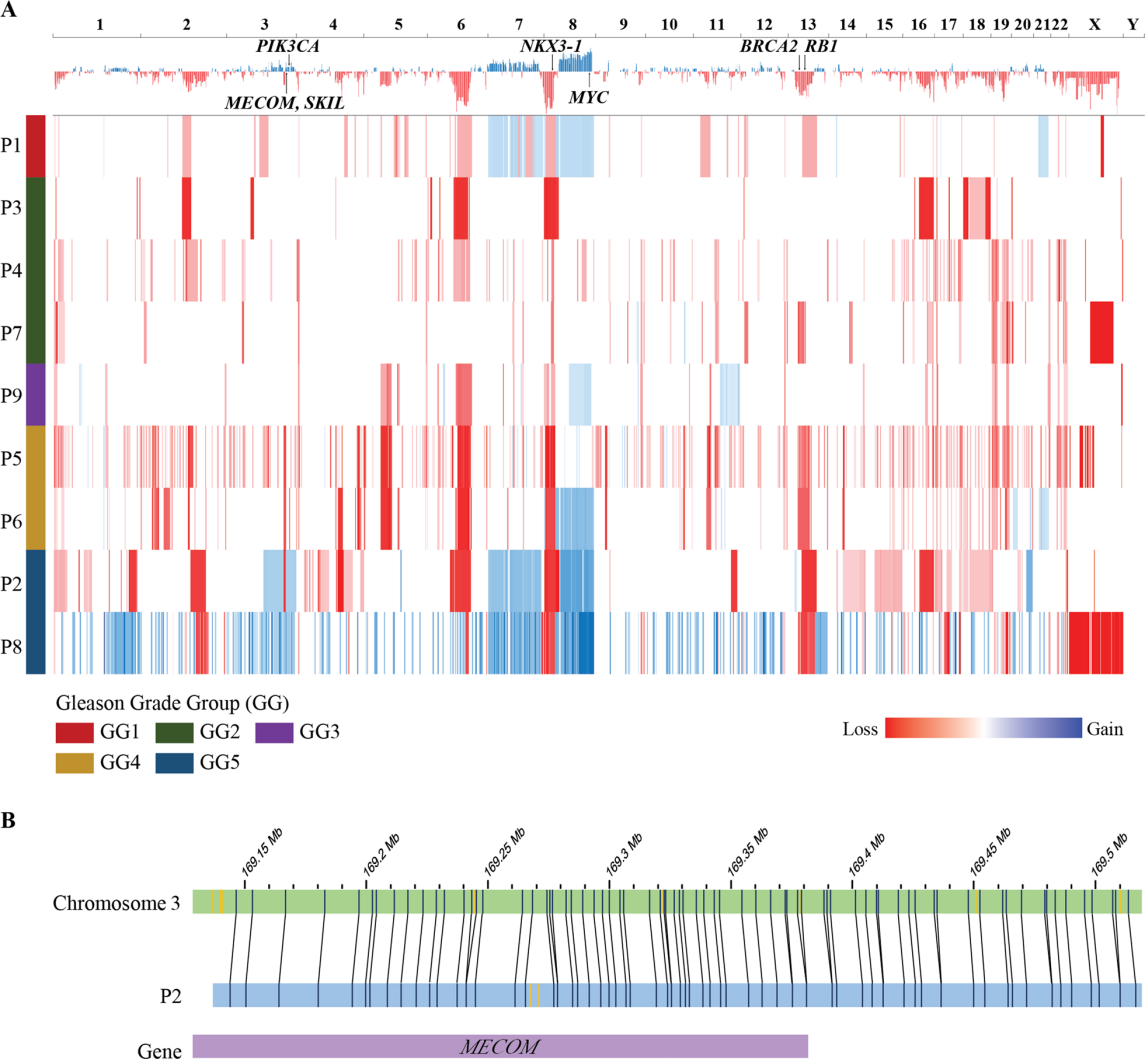
Copy Number Variations of Prostate Cancer Tissue Sample. **A** Copy number variation landscape of 9 samples. **B** Insertion at *MECOM* gene, chr3q26 of sample P2. The location of *MECOM* gene is displayed as purple bar. De novo genome maps (blue) are aligned to the reference genome (green). The labeled specific sequence motifs are shown as black vertical lines on each bar. The black lines indicate the alignment between the reference and assembled map. The yellow vertical lines indicate additional labels

For copy number alteration, chromosome (Chr) 6q13 loss and Chr8q12-24 gain were the most frequent events (78% and 44%, respectively). Chr8q12-24 gain (harboring *MYC* oncogene) was frequently accompanied by Chr8p21-23 loss (harboring *NKX3-1* tumor suppressor gene) (Fig. 2A). Loss of Chr13q13.3-q22.2 (harboring tumor suppressor *RB1*) and gain of Chr3q26 was observed in two GG 5 samples (P2 and P8) (Fig. 2A). Notably, the gain of Chr3q26 in P2 sample was accompanied by insertion in a histone-lysine N-methyltransferase gene *MECOM* (Fig. 2B). A 2.4 Mb-size deletion Chr17p21 involving *BRCA1* was observed in one GG 2 sample (P3).

**Fig. 3:**
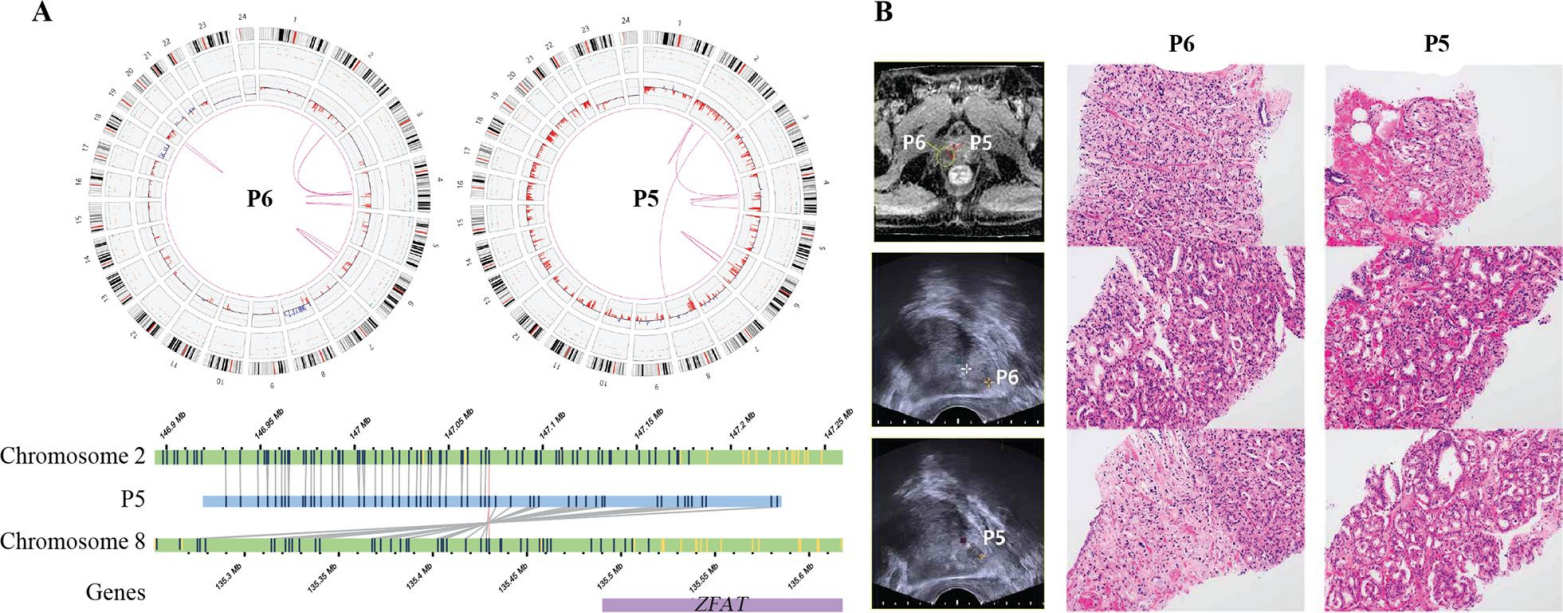
Intratumoral Heterogeneity and Complex Genomic Rearrangement. **A** Intratumoral genomic heterogeneity. P6 and P5 were from same patient. **B** MRI, US images and representative H&E staining images of P6 and P5. P5: The majority of tumor was composed of atypical tumor glands with glomeruloid feature, which is compatible with Gleason grade 4. As the second most common component, Gleason grade 3 tumor was identified. P6: On the contrary to P5 tumor, the majority of P6 tumor was comprised of atypical fused glands, showing Gleason pattern 4 (upper). Similar to P5 tumor, Gleason grade 4 tumors with glomeruloid feature was identified and admixed with Gleason grade 3 tumor, the second most common component (middle). A few scattered single tumor cells, which is compatible with Gleason grade 5 were identified as the third pattern (lower)

We further analyzed inter- and intra-chromosomal translocations (Additional file 1: Fig. S1). We focused P5 and P6, which were derived from two adjacent tumors identified by MRI in a single patient (Fig. 3A). While the two samples shared some intra-chromosomal translocations, they were differed from each other by additional inter or intra-chromosomal translocations, of which breakpoints are occurring at the shared translocation sites. For example, both P5 and P6 exhibited t(4;4)(q22.2:q25), but only P6 had t(2;4)(p11.3:q25) and t(2;4)(q32.1:q25). In contrast, P5 had t(2;4)(p16,3:q22.3). Similarly, both P5 and P6 exhibited t(6;6)(q15:q22.31), but only P6 had t(6;6)(q12:q22.31), and only P5 had t(6;6)(q14.3:q22.31). Furthermore, t(2;8)(q22.3:q24.22) was observed only in P5. For other types of SVs, most indels found in P6 overlapped with P5. The total number of SVs was 895 in P6 and 899 in P5 (Table 2). While both P5 and P6 were graded as GG4, we observed difference of dominant histological pattern between the tumors (Fig. 3B).

In sample P9, a GG 3 tumor described as PI-RAD5 region in MRI, OGM found deletion of chr9p23 (start position: 10,018,386; end position: 10,022,066) affecting *PTPRD* gene. Further panel sequencing (Trusight™ Oncology kit, Illumina) found a frameshift mutation of *PTPRD* (AA change: p.T1578Lfs*2; HGV Sc: NM_002839.3:c.4732delA).

**Fig. 4:**
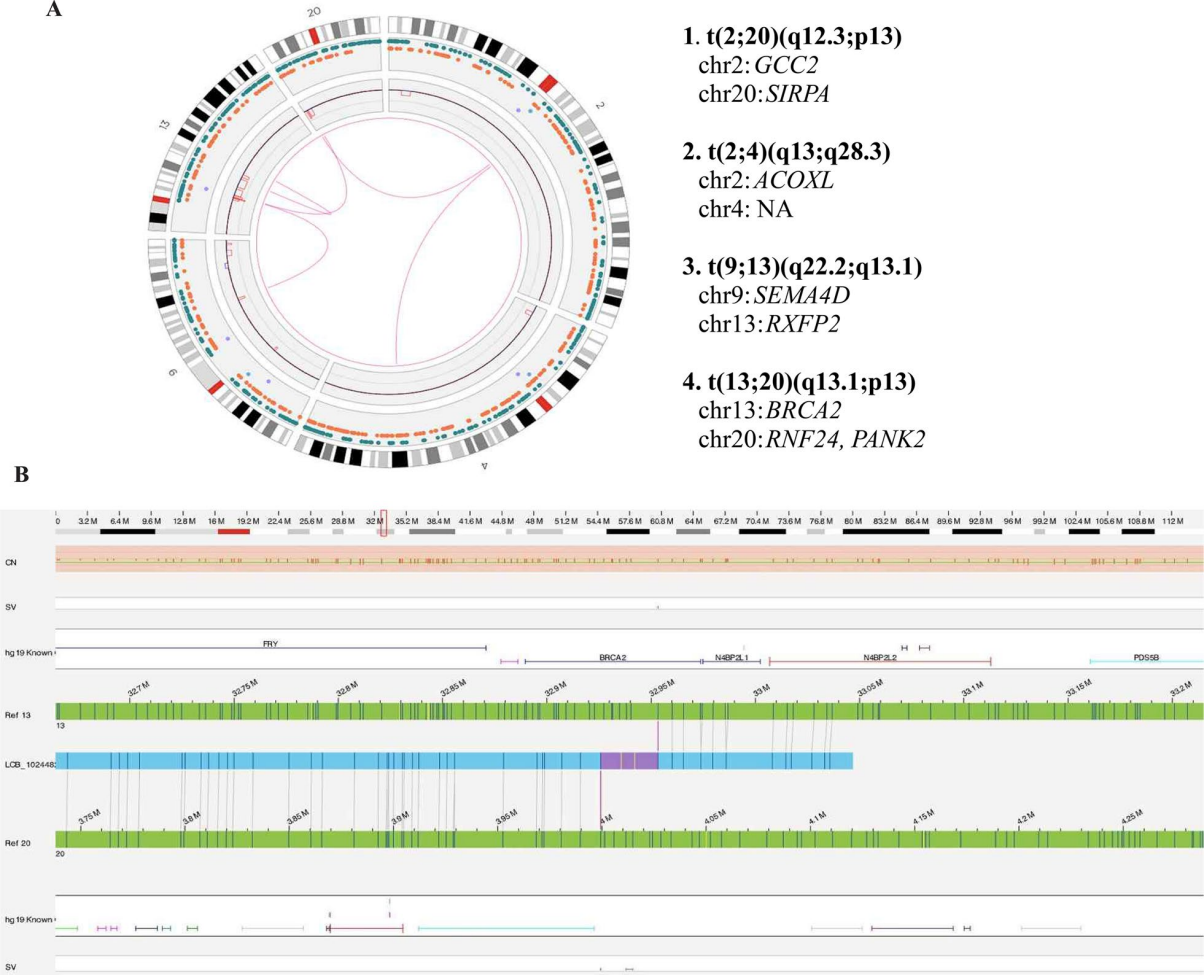
Complex Genomic Rearrangements involving *BRCA2.* **A** Circos plot showing multiple translocations and copy number variations of P8. Translocations locus described in right. **B** Chromosome view of t(13;20)(q13.1;p13)

Lastly, we describe a complex genomic rearrangement affecting *BRCA2* at P8, a GG 5 tumor. One copy of *BRCA2* was lost by wide copy number loss of chr13q12.3-14.3, also involving *RB1* (Fig. 4A). The other copy of *BRCA2* was disrupted by an inter-chromosomal translocation t(13;20)(q13.1;p13) (Fig. 4B). Interestingly, multiple inter or intra-chromosomal translocations occurred nearby the translocation breakpoints - t(2;20)(q12.3;p13) involving *GCC2* and *SIRPA*, t(2;4)(q13;q28.3) involving *ACOXL*, and t(9;13)(q22.2;q13.1) involving *SEMA4D* and *RXFP2* (Fig. 4A). Importantly, this patient had a history of malignant choroidal melanoma. We performed germline panel sequencing, which did not reveal relevant mutations related to cancer susceptibility. Peripheral blood germline testing of t(13;20)(q13.1;p13) is planned. Additional chromosomal translocation data are summarized in Additional file 2: Table S3.

## Discussion

In this study, we aimed to assess the utility of OGM to detect complex genomic rearrangements using consecutive unselected series of PCa samples via MRI/US fusion biopsy. Gleason grade group (GG) is a well-established prognostic indicator of PCa retrieved from biopsy. High GG (4–5) tumors tended to show more frequent duplications, inversions, and translocations than low GG (1–3) tumors. It has been known that the burdens of genomic SVs (copy number alterations, aneuploidy) correlate with Gleason grades and prognosis [4, 17, 18].

Compared to the other cancers, PCa genome is characterized by frequently balanced rearrangements that affect multiple oncogenes and tumor suppressor genes [19]. While most SVs involved intergenic or intronic noncoding genomic regions, we identified SVs that affect oncogenes (*MYC* and *MECOM*) and tumor suppressor genes (*NKX3-1*, *RB1*, *BRCA1*, *BRCA2*, and *PTPRD*). *PTPRD* encodes protein tyrosine phosphatase and is frequently inactivated in solid tumors such as glioblastoma [20]. We showed that a case of *PTPRD* truncating mutation co-occurs with the allelic loss of the *PTPRD* gene. *BRCA2* is one of the most frequently mutated DNA repair genes in advanced PCa [21]. About 1.1% of unselected PCa had germline *BRCA2* mutations, and about 4.9% had somatic mutations [22]. Detection of *BRCA2* genetic alteration is clinically relevant because it suggests a response to PARP inhibitors [8, 9], and familial analysis is indicated if germline mutation is suspected. Somatic alterations from large genomic rearrangements are frequent in *BRCA1/2* due to genomic instability from high densities of repetitive elements in the *BRCA1/2* genes [23]. In our case, single copy loss of *BRCA2* and *RB1* by large deletion of chromosome 13q12-14 and inter-chromosomal translocation of breakpoint occurring at *BRCA2* was found. Molecular studies suggest that *BRCA2* mutation combined with *RB1* alteration, located on chromosome 13q14, can generate an aggressive PCa model with castration resistance [24]. In particular, in the case of balanced translocation that involves *BRCA2*, like our case, detection is unavailable with CMA, and it is challenging to detect short-read sequencing.

We observed a split duplication involving *TMPRSS2*, but no other ETS family gene rearrangement was found (Additional file 2: Table S3). ETS genomic rearrangement frequency varies across races, and in the Asian population, the frequency is lowest. Li et al. analyzed 208 primary prostate cancer tissues by WGS and found that only 9% of the tumors had *TMPRSS2*-*ERG* fusion [25]. In their analysis, *FOXA1* and *SPOP* mutations were more frequent than those reported in western populations, which are known to be mutually exclusive to ETS genomic rearrangements.

This study has several limitations. First, due to a relatively small number of subjects included in this study, sufficient power was not achieved to access the association between identified SVs and clinical features. Second, we did not analyze patients’ follow-up data because we used diagnostic PCa samples prospectively. Third, we did not confirm the identified SVs with another assay such as WGS. While we did not incorporate WGS in our study, earlier studies combining OGM and WGS in advanced PCa samples have found that large insertions and duplications were frequently missed by short-read WGS discovery approach [14, 26]. In hematologic malignancies, studies comparing OGM to conventional cytogenetic approaches also reported that OGMs identified large (> 5 Mb) or complex SVs that were not detected by conventional tools [16, 27]. For PCa, a more extensive study with long-term follow-up data is necessary to confirm the clinical implication of those large or complex SVs.

OGM has the advantage of detecting complex structural rearrangements. However, OGM alone is not sufficient for confirming the exact rearrangement position. As OGM compares motif-specific label patterns to the reference genome instead of sequencing base pairs, the genomic location of the variants is not accurate [10]. We previously observed location differences between OGM and chromosomal microarray. Therefore, the exact rearrangement position detected by OGM needs to be confirmed by sanger sequencing or NGS yet.

## Conclusion

In conclusion, OGM represents a promising tool identifying SVs disrupting PCa relevant genes with clinical and therapeutic significance. All consecutive biopsy samples passed quality checks except for one, and we successfully detected SVs in all tested samples. We suggest that OGM can complement short-read sequencing in cancer molecular assessment.

## Supplementary Information

**Additional file 1: Fig. S1.** Inter/Intra-chromosomal translocations.

**Additional file 2: Table S1.** Genome mapping summary. **Table S2.** All non-masked CNVs called by Bionano Analysis Pipeline. **Table S3.** All SVs called by Bionano Analysis Pipeline.

## Data Availability

All data generated or analyzed during this study are included in this published article and its supplementary information files.

## Abbreviations

PCa: Prostate cancer
SV: Structural variation
FISH: Fluorescence in situ hybridization
CMA: Chromosomal microarrays
WGS: Whole-genome sequencing
OGM: Optical genome mapping
US: Ultrasonography
UHMW: Ultra-high molecular weight
DLS: Direct label and staining
GG: Gleason grade group
MRI: Magnetic resonance imaging
PSA: Prostate specific antigen
ISUP GG: International society of urological pathology grade group
TZ: Transition zone
PI-RADS: Prostate imaging–reporting and data system
PZ: Peripheral zone
Chr: Chromosome

## References

[1] Baca SC, Prandi D, Lawrence MS, Mosquera JM, Romanel A, Drier Y (2013). Punctuated evolution of prostate cancer genomes. Cell.

[2] Li Y, Roberts ND, Wala JA, Shapira O, Schumacher SE, Kumar K (2020). Patterns of somatic structural variation in human cancer genomes. Nature.

[3] Gasi Tandefelt D, Boormans J, Hermans K, Trapman J (2014). ETS fusion genes in prostate cancer. Endocr Relat Cancer.

[4] Quigley DA, Dang HX, Zhao SG, Lloyd P, Aggarwal R, Alumkal JJ (2018). Genomic hallmarks and structural variation in metastatic prostate cancer. Cell.

[5] Castro E, Goh C, Olmos D, Saunders E, Leongamornlert D, Tymrakiewicz M (2013). Germline BRCA mutations are associated with higher risk of nodal involvement, distant metastasis, and poor survival outcomes in prostate cancer. J Clin Oncol.

[6] Wei Y, Wu J, Gu W, Wang J, Lin G, Qin X (2020). Prognostic value of germline DNA repair gene mutations in de novo metastatic and castration-sensitive prostate cancer. Oncologist.

[7] Annala M, Vandekerkhove G, Khalaf D, Taavitsainen S, Beja K, Warner EW (2018). Circulating Tumor DNA Genomics Correlate with Resistance to Abiraterone and Enzalutamide in Prostate Cancer. Cancer Discov.

[8] de Bono J, Mateo J, Fizazi K, Saad F, Shore N, Sandhu S (2020). Olaparib for metastatic castration-resistant prostate cancer. N Engl J Med.

[9] Abida W, Patnaik A, Campbell D, Shapiro J, Bryce AH, McDermott R (2020). Rucaparib in men with metastatic castration-resistant prostate cancer harboring a BRCA1 or BRCA2 gene alteration. J Clin Oncol.

[10] Lowrance WT, Breau RH, Chou R, Chapin BF, Crispino T, Dreicer R (2021). Advanced Prostate Cancer: AUA/ASTRO/SUO Guideline PART II. J Urol.

[11] Mantere T, Neveling K, Pebrel-Richard C, Benoist M, van der Zande G, Kater-Baats E (2021). Optical genome mapping enables constitutional chromosomal aberration detection. Am J Hum Genet.

[12] Chan EKF, Cameron DL, Petersen DC, Lyons RJ, Baldi BF, Papenfuss AT (2018). Optical mapping reveals a higher level of genomic architecture of chained fusions in cancer. Genome Res.

[13] Goldrich DY, LaBarge B, Chartrand S, Zhang LJ, Sadowski HB, Zhang Y (2021). Identification of somatic structural variants in solid tumors by optical genome mapping. J Personalized Med.

[14] Crumbaker M, Chan EKF, Gong TT, Corcoran N, Jaratlerdsiri W, Lyons RJ (2020). The impact of whole genome data on therapeutic decision-making in metastatic prostate cancer: a retrospective analysis. Cancers.

[15] Lestringant V, Duployez N, Penther D, Luquet I, Derrieux C, Lutun A (2021). Optical genome mapping, a promising alternative to gold standard cytogenetic approaches in a series of acute lymphoblastic leukemias. Genes Chromosomes Cancer.

[16] Kriegova E, Fillerova R, Minarik J, Savara J, Manakova J, Petrackova A (2021). Whole-genome optical mapping of bone-marrow myeloma cells reveals association of extramedullary multiple myeloma with chromosome 1 abnormalities. Sci Rep.

[17] Stopsack KH, Whittaker CA, Gerke TA, Loda M, Kantoff PW, Mucci LA (2019). Aneuploidy drives lethal progression in prostate cancer. Proc Natl Acad Sci U S A.

[18] Hieronymus H, Schultz N, Gopalan A, Carver BS, Chang MT, Xiao Y (2014). Copy number alteration burden predicts prostate cancer relapse. Proc Natl Acad Sci U S A.

[19] Berger MF, Lawrence MS, Demichelis F, Drier Y, Cibulskis K, Sivachenko AY (2011). The genomic complexity of primary human prostate cancer. Nature.

[20] Veeriah S, Brennan C, Meng S, Singh B, Fagin JA, Solit DB (2009). The tyrosine phosphatase PTPRD is a tumor suppressor that is frequently inactivated and mutated in glioblastoma and other human cancers. Proc Natl Acad Sci U S A.

[21] Pritchard CC, Mateo J, Walsh MF, De Sarkar N, Abida W, Beltran H (2016). Inherited DNA-repair gene mutations in men with metastatic prostate cancer. N Engl J Med.

[22] Lang SH, Swift SL, White H, Misso K, Kleijnen J, Quek RGW (2019). A systematic review of the prevalence of DNA damage response gene mutations in prostate cancer. Int J Oncol.

[23] Welcsh PL, King MC (2001). BRCA1 and BRCA2 and the genetics of breast and ovarian cancer. Hum Mol Genet.

[24] Chakraborty G, Armenia J, Mazzu YZ, Nandakumar S, Stopsack KH, Atiq MO (2020). Significance of BRCA2 and RB1 co-loss in aggressive prostate cancer progression. Clin Cancer Res.

[25] Li J, Xu C, Lee HJ, Ren S, Zi X, Zhang Z (2020). A genomic and epigenomic atlas of prostate cancer in Asian populations. Nature.

[26] Jaratlerdsiri W, Chan EKF, Petersen DC, Yang C, Croucher PI, Bornman MSR (2017). Next generation mapping reveals novel large genomic rearrangements in prostate cancer. Oncotarget.

[27] Neveling K, Mantere T, Vermeulen S, Oorsprong M, van Beek R, Kater-Baats E (2021). Next-generation cytogenetics: comprehensive assessment of 52 hematological malignancy genomes by optical genome mapping. Am J Hum Genet.

