# Supplementary figures and images for: Optical genome mapping identifies clinically relevant genomic rearrangements in prostate cancer biopsy sample
Source: Cancer Cell Int. 2022 Oct 8;22:306. doi: 10.1186/s12935-022-02728-2 (PMC9548106; doi:10.1186/s12935-022-02728-2)

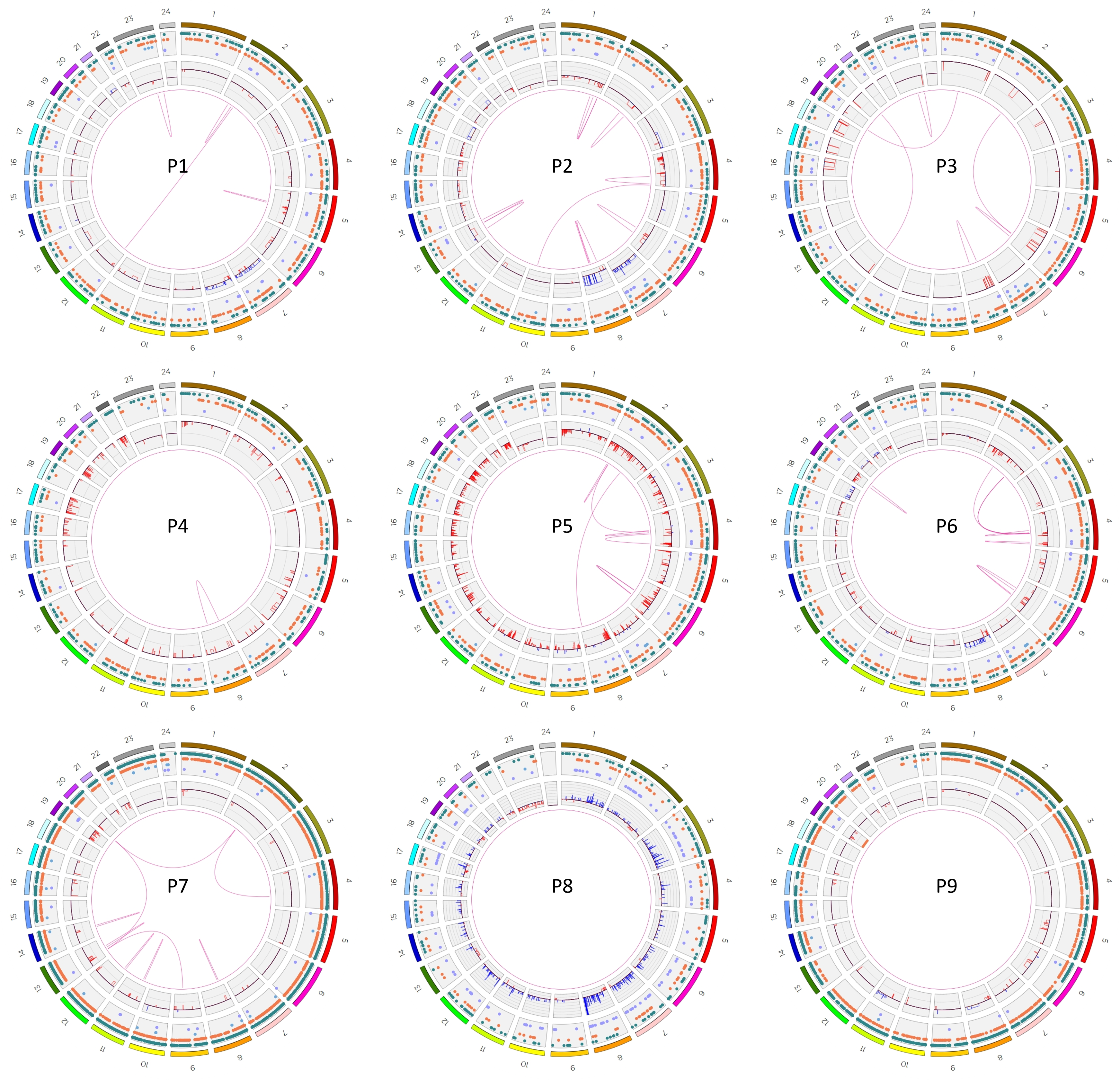

Supplement: Supplementary file 1 — Additional file 1: Fig. S1. Inter/Intra-chromosomal translocations. [file 12935_2022_2728_MOESM1_ESM.png]
